# A novel strategy for creating a new system of third‐generation hybrid rice technology using a cytoplasmic sterility gene and a genic male‐sterile gene

**DOI:** 10.1111/pbi.13457

**Published:** 2020-08-27

**Authors:** Shufeng Song, Tiankang Wang, Yixing Li, Jun Hu, Ruifeng Kan, Mudan Qiu, Yingde Deng, Peixun Liu, Licheng Zhang, Hao Dong, Chengxia Li, Dong Yu, Xinqi Li, Dingyang Yuan, Longping Yuan, Li Li

**Affiliations:** ^1^ State Key Laboratory of Hybrid Rice Hunan Hybrid Rice Research Center Hunan Academy of Agricultural Sciences Changsha China; ^2^ State Key Laboratory of Hybrid Rice Engineering Research Center for Plant Biotechnology and Germplasm Utilization of Ministry of Education College of Life Sciences Wuhan University Wuhan China; ^3^ State Key Laboratory of Applied Optics Changchun Institute of Optics Fine Mechanics & Physics Chinese Academy of Sciences Changchun China; ^4^ College of Agronomy Hunan Agricultural University Changsha China; ^5^ Long Ping Branch Graduate School of Hunan University Changsha China

**Keywords:** Breeding technology, *CYP703A3*, *DsRed2*, *orfH79*, third‐generation hybrid rice technology

## Abstract

Heterosis utilization is the most effective way to improve rice yields. The cytoplasmic male‐sterility (CMS) and photoperiod/thermosensitive genic male‐sterility (PTGMS) systems have been widely used in rice production. However, the rate of resource utilization for the CMS system hybrid rice is low, and the hybrid seed production for the PTGMS system is affected by the environment. The technical limitations of these two breeding methods restrict the rapid development of hybrid rice. The advantages of the genic male‐sterility (GMS) rice, such as stable sterility and free combination, can fill the gaps of the first two generations of hybrid rice technology. At present, the third‐generation hybrid rice breeding technology is being used to realize the application of GMS materials in hybrid rice. This study aimed to use an artificial CMS gene as a pollen killer to create a smart sterile line for hybrid rice production. The clustered regularly interspaced short palindromic repeats/CRISPR‐associated 9 (CRISPR/Cas9) technology was used to successfully obtain a *CYP703A3*‐deficient male‐sterile mutant containing no genetically modified component in the genetic background of indica 9311. Through young ear callus transformation, this mutant was transformed with three sets of element‐linked expression vectors, including pollen fertility restoration gene *CYP703A3*, pollen‐lethality gene *orfH79* and selection marker gene *DsRed2*. The maintainer 9311‐3B with stable inheritance was obtained, which could realize the batch breeding of GMS materials. Further, the sterile line 9311‐3A and restorer lines were used for hybridization, and a batch of superior combinations of hybrid rice was obtained.

## Introduction

The global agricultural production must increase by 70% in the next 30 years to feed the world’s population, which will increase from almost 7.6 billion to an estimated more than 9.8 billion in 2050 (Carvajal‐Yepes *et al*., 2019). Meanwhile, plant diseases, crop insects, drought and environmental deterioration due to climate change greatly impair crop production and sustainable development (Bhattacharya, 2017; Guo *et al*., 2018; Hu *et al*., 2013). Therefore, food security is the most critical problem affecting mankind. The population increase mainly affects the developing countries, especially Asia. Rice is cultivated in more than 100 countries with 90% of the total global production in Asian countries (Fukagawa and Ziska, 2019), where it represents the main staple food resource. Obviously, increasing rice production is one of the most efficient ways to ensure food security in the future.

Technological advances in the agricultural sector, especially plant breeding techniques, contributed to an increase in agricultural productivity (Du *et al*., 2020; Gils *et al*., 2008; Khan *et al*., 2019; Ray *et al*., 2007). In the last 50 years, remarkable growth in agricultural production has largely depended on cereal crops, especially rice, in China (Fan *et al*., 2012). Two great leaps in rice production were the use of semi‐dwarf gene and heterosis from hybrid rice (Zhang, 2007). At present, the main use of heterosis from hybrid rice includes CMS and PTGMS systems in China (Chen and Liu, 2014; Gu *et al*., 2019; Zhang, 2007). Several CMS genes have been identified as aberrant, often chimeric, mitochondrial genes in sterile lines, which can be suppressed by Rf genes from restorer lines (Hu *et al*., 2014). Despite the stability and complete sterility of the CMS line, which is safe in hybrid seed production, the application of CMS/Rf system in breeding is limited by the need for restorer genes, which compensates the CMS‐caused impairment in F1 hybrids. As the previously characterized PTGMS genes are nuclear‐recessive and male fertility can be restored by any other normal rice cultivars (Ding *et al*., 2012; Zhou *et al*., 2012), the system can contribute to a broader exploration of heterosis. However, the fertility of PTGMS lines is greatly affected by the external environment, making hybrid seed production quite vulnerable (Tao *et al*., 2003). Therefore, generating new sterile lines capable of safe hybrid seed production and amenable to free combination for the development of hybrid rice is imperative.

The advantages of genic male‐sterile rice, including stable sterility, safe hybrid seed production and free combination, can fill the gaps of the first two generations of hybrid rice technology, but is insufficient for the batch breeding of sterile line seeds, severely blocking its widespread adoption. Previous studies proposed a series of solutions to respond to the need for the breeding of nuclear sterile materials. In 1993, the Plant Genetic Systems Company proposed the generation of a maintainer line of male‐sterile plants through the expression in plants of three sets of element‐linked vectors, including fertility restoration gene, pollen abortion gene and selection marker gene. In this scenario, the breeding of sterile lines was achieved by self‐crossing. In 2002, Perez‐Prat et al. proposed two sets of element systems in which fertility restoration gene and selection marker gene linkages were expressed in male‐sterile mutants to achieve the breeding of sterile lines (Perez‐Prat *et al*., 2002). In 2006, the US DuPont–Pioneer Company realized the seed production technology (SPT) of GMS in maize for the first time (Albertsen *et al*., 2006). In 2013, Deng et al. mentioned that new hybrid seed production systems using modern biotechnology and molecular crop design might lead to a new era of hybrid rice practice (Deng *et al*., 2013). In 2016, Yuan Longping pointed out that the preliminary study on the third‐generation hybrid rice technology was successful, and it involved hybrid rice with a genetically engineered male‐sterile line as a genetic tool. The genic sterile rice was bred into a genetically engineered male‐sterile line by genetic engineering technology. It combined the advantages of the stable sterility of the CMS system and the free combination of the PTGMS system (Yuan, 2016). In 2016, previous studies used the rice fertility gene *OsNP1*, pollen inactivation gene *ZmAA1* and red fluorescent protein (RFP) gene linkages expressed in the rice pollen‐free nuclear sterile mutant *osnp1‐1*, achieving the breeding of the genic male‐sterile line and creating a genetically stable sterile line, Zhen18A and corresponding maintainer line (Zhen18B) (Chang *et al*., 2016). Recently, another group also used *ZmMs7*, *ZmAA1* and *DsRed2* to construct a sterile line in maize for hybrid seed production (Zhang *et al*., 2018). Up to now, *ZmAA1* and *Dam* were reported to be used as a pollen killer in both of the aforementioned two systems (Wang *et al*., 2020; Zhang *et al*., 2018). Relative to the abundance of GMS genes, exploring other pollen‐killer genes in plants is critical.

In this study, the third‐generation hybrid rice technology system was upgraded with a novel strategy, which used a CMS gene as a pollen killer. *CYP703A3* (Yang *et al*., 2014; Yang *et al*., 2018) was knocked out by the CRISPR/Cas9 technology to obtain the *CYP703A3* loss‐of‐function mutant *9311^03a3^* without foreign transgenic vectors. Furthermore, the CMS gene, *orfH79* (GenBank accession numbers: KC188738; Peng, *et al*., 2010), from Honglian CMS (HL‐CMS) rice, was used to verify the pollen inactivation system. The artificial ORFH79 fused with *RF1b* (Wang *et al*., 2006) mitochondrial signal peptide (MSP) driven by the pollen‐specific promoter *PG47* (Allen and Lonsdale, 1993) rendered infertile the pollen of indica rice cultivar 9311. Subsequently, the cassette of the recombinant vector, containing *CYP703A3*, *DsRed2* and *orfH79*, was introduced into mutant *9311^03a3^* to maintain the sterility. Moreover, a mechanized sorting system was successfully developed for 9311‐3A and 9311‐3B seed production. In addition, the sterile line 9311‐3A was crossed with several cultivars to evaluate heterosis. In conclusion, this study was novel in using an artificial CMS gene as a pollen killer to create the third‐generation hybrid rice more efficiently and demonstrating that more mitochondrial chimeric genes (including orfs) could be applied to the generation of pollen‐killer systems.

## Results

### Construction of the *CYP703A3* knockout mutants

A previous study reported that *CYP703A3* encoded a cytochrome P450 hydroxylase and was critical for pollen development (Yang *et al*., 2014; Yang *et al*., 2018). Therefore, *CYP703A3* was selected as the target gene to create the male‐sterile line. For the genetic background, line 9311 was chosen, which was one of the most widely used elite lines in hybrid rice in the last 20 years. The CRISPR/Cas9 technology was used to obtain the line with the loss of function of *CYP703A3*.

**Figure 1:**
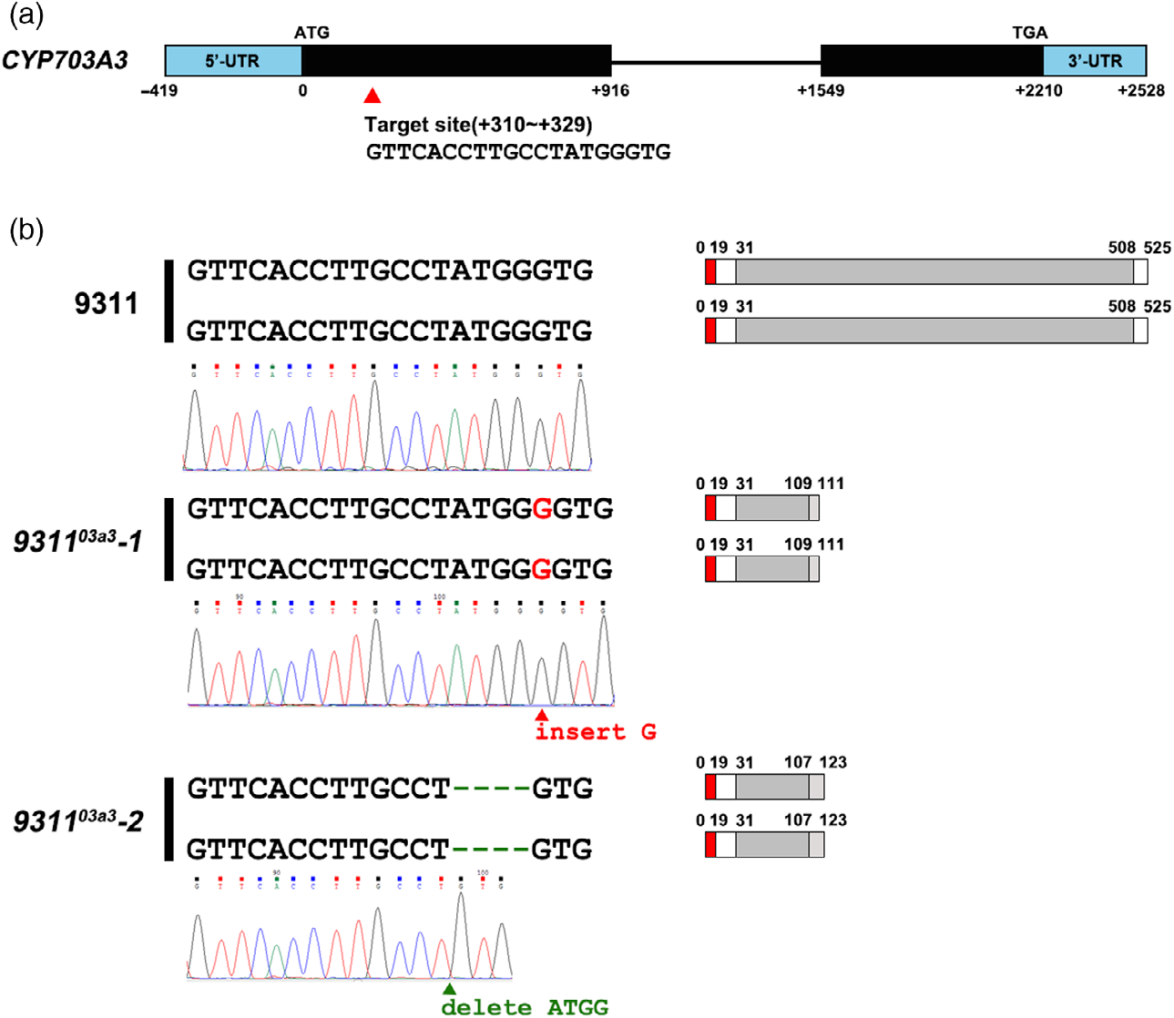
Analysis of the mutation type of the rice *CYP703A3* loss‐of‐function mutant *9311^03a3^*. (a) Schematic diagram of the *CYP703A3* gene structure. The red triangle indicates the gene‐editing targeting site on *CYP703A3*. (b) Mutation types and types of the encoded protein of the mutant *9311^03a3^*. The red region shows the signal peptide, and the dark grey region indicates the P450 domain. [Colour figure can be viewed at wileyonlinelibrary.com]

*CYP703A3* consisted of two exons with a full sequence length of 2210 bp encoding a P450 core domain comprising 478 amino acids. In this study, the targeted editing site was located at positions 310–329 nt (Figure 1a and Figure S1). Two independent *CYP703A3* knockout mutants were obtained, namely *9311^03a3^‐1* and *9311^03a3^‐2*. In the *9311^03a3^‐1* mutant, a G base was inserted after position 326, resulting in a frameshift and a premature stop codon. The resulting coding sequence (CDS) encoded a peptide comprising 11 amino acid residues (with only 79 residues remaining of the P450 domain). In the *9311^03a3^‐2* mutant, the deletion of the ATGG sequence at 323 nt also led to a frameshift and a premature stop codon (Figure 1b). The corresponding predicted peptide was 123‐amino acid residue long (with only 77 amino acids remaining of the P450 domain).

### Phenotypic analysis of the male‐sterile mutant *9311^03a3^*

**Figure 2:**
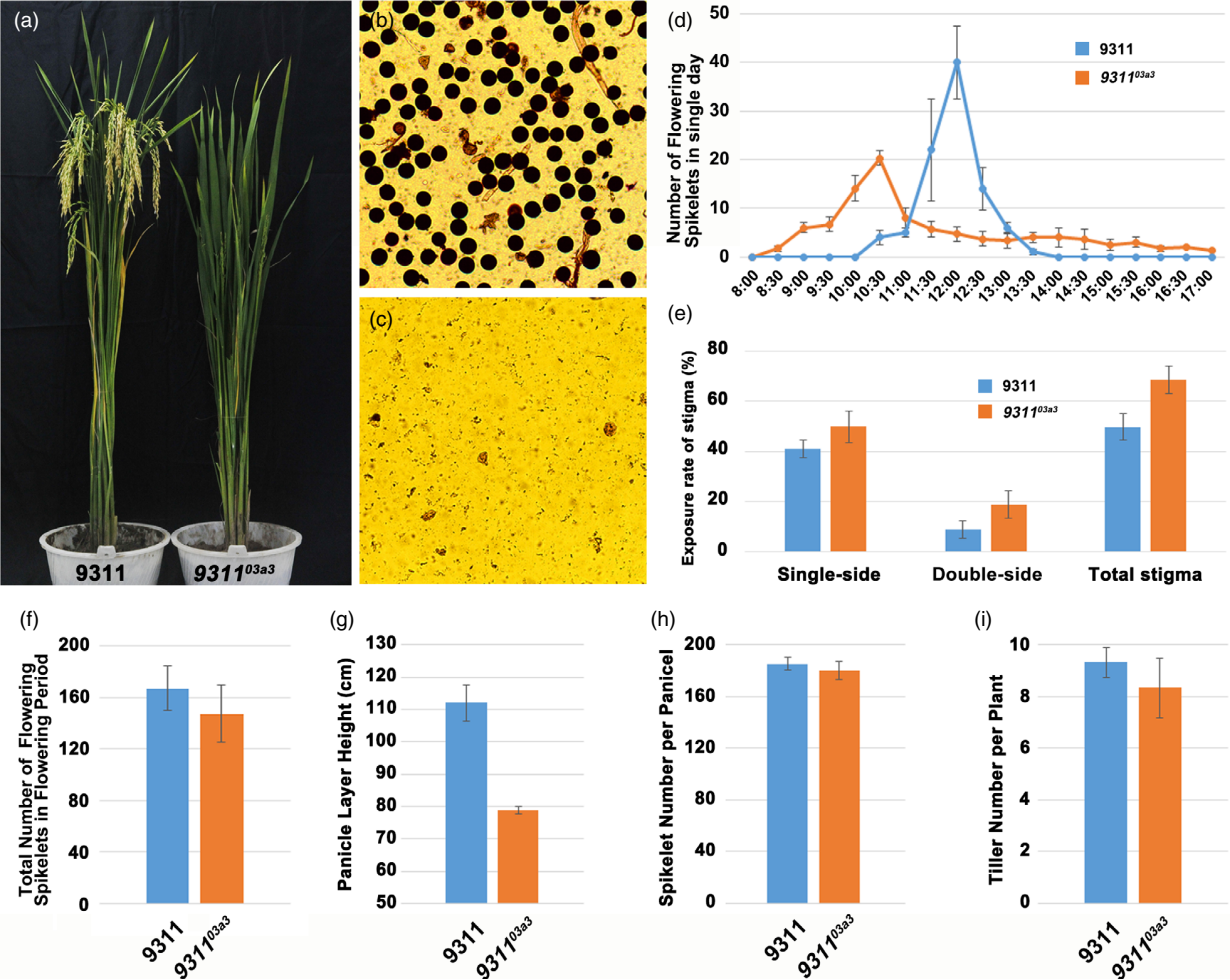
Analysis of phenotype and flowering habits of the male‐sterile mutant *9311^03a3^*. (a) Observation of the plant architecture of wild‐type 9311 and mutant *9311^03a3^*. (b, c) Pollen fertility testing of wild‐type 9311 and mutant *9311^03a3^*. (d) The daily flowering dynamic survey in wild‐type 9311 and mutant *9311^03a3^*. (e) Statistics on the stigma exsertion rate of a single panicle of wild‐type 9311 and mutant *9311^03a3^*. (f) Statistics on the total number of flowering spikelets of single panicle of 9311 and *9311^03a3^* during the flowering period. (g–i) Statistics on the panicle layer height, total spikelet number per panicle and tiller number per plant of wild‐type 9311 and mutant *9311^03a3^*. [Colour figure can be viewed at wileyonlinelibrary.com]

The phenotypes of these mutants were characterized in detail to ensure that the mutants could be used in the future. The mutant *9311^03a3^* was completely sterile, the amount of pollen of *9311^03a3^* was dramatically reduced, and only a few shrivelled sterile pollen grains were observed (Figure 2a–c). This was consistent with a previous study showing that loss of function of *CYP703A3* led to complete pollen abortion in the variety 9311, in which complete restoration of fertility occurred by complementation (Figure S2). Next, the flowering time and the number of flowering spikelets of this mutant line were checked as flowering characters were important for sterile lines. Statistical analysis showed that the flowering time of *9311^03a3^* (typically from 9:30 to 11:00) was earlier than that of the wild‐type 9311 (typically from 11:00 to 12:30; Figure 2d). The total flowering spikelet number per panicle between *9311^03a3^* and 9311 had no obvious difference (Figure 2f). Stigma is quite important for outcrossing, which is essential for hybrid production. Therefore, the exsertion rate of stigma was statistically compared between *9311^03a3^* and wild‐type 9311 in this study. The results revealed that the exsertion rates of the unilateral, bilateral and total stigma in *9311^03a3^* were higher than those in 9311 (Figure 2e), and the outcrossing rate of *9311^03a3^* was 58.7%. Furthermore, the panicle layer height of *9311^03a3^* and 9311 was also measured. The results also showed that the mutant *9311^03a3^* was lower than 9311(Figure 2g). Subsequently, the spikelet number and the tiller number, traits that also contribute to seed production, were compared between *9311^03a3^* and 9311. No obvious differences in these two traits were observed, consistent with a previous study (Figure 2h,i). In conclusion, a novel elite male‐sterile line was successfully created by knocking out *CYP703A3* in the 9311 background, with the resulting mutant line *9311^03a3^* exhibiting favourable performance for hybrid seed production.

### Construction of a pollen lethal system using *orfH79*

**Figure 3:**
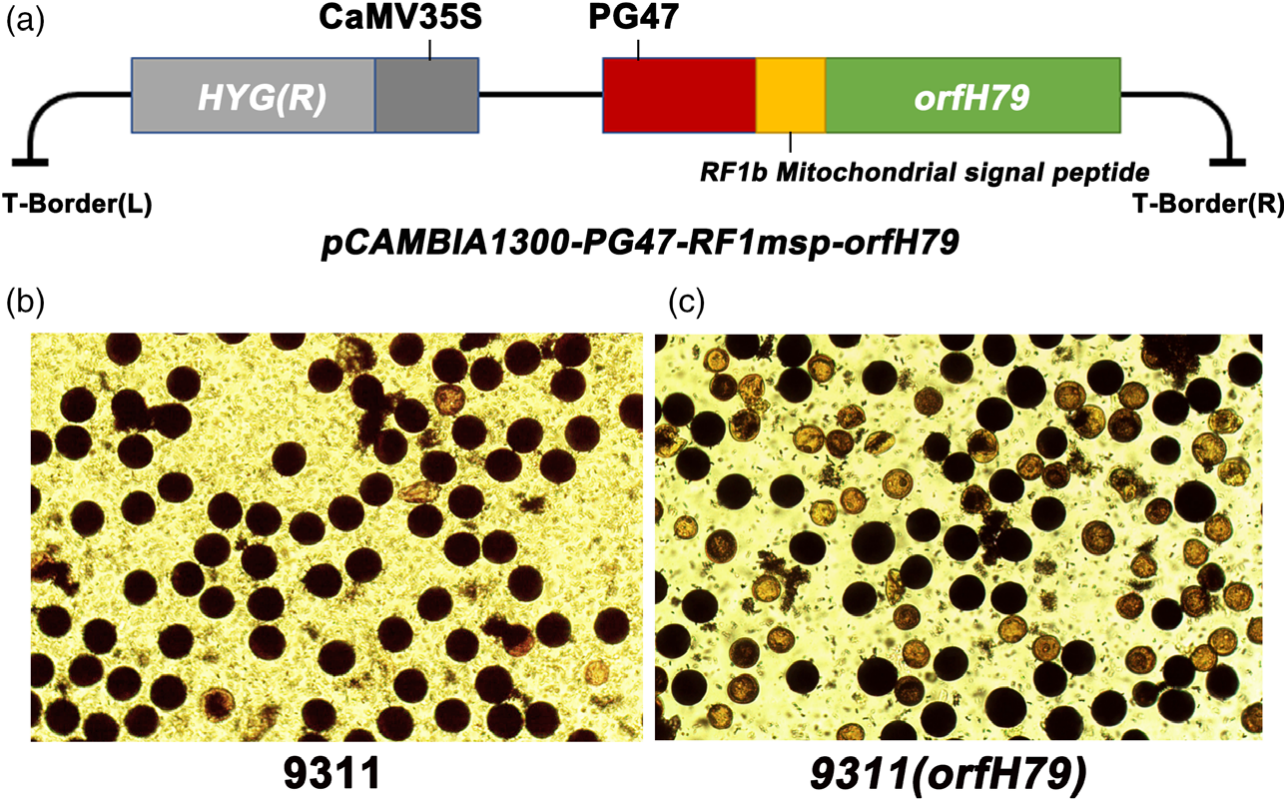
Construction of the pollen inactivation vector and fertility testing. (a) Schematic diagram of the pollen inactivation vector. (b, c) Pollen fertility testing of wild‐type 9311 and *9311* (*orfH79*). [Colour figure can be viewed at wileyonlinelibrary.com]

A previous study confirmed that the HL‐CMS gene, *orfH79*, could lead to gametophytic male sterility, suggesting that the male gametes could be killed via the ectopic expression of cytotoxic ORFH79 in pollen (Peng, *et al*., 2010). An expression vector including the pollen‐specific promoter *PG47* was employed to verify the effectiveness of *orfH79* for rice pollen inactivation. Considering that the cytotoxic ORFH79 functions in mitochondria and interacts with P61, which is a subunit of complex III of electron transport chain (Wang, *et al*., 2013), the *RF1b* MSP was fused at the N‐terminus of ORFH79 (Figure 3a). The construct was used to obtain independent transgenic plants in 9311 genetic background, *9311* (*orfH79*). Pollen fertility testing of the transgenic plants revealed that the *9311* (*orfH79*) pollen grains were semi‐dysfunctional (sterility:fertile = 148:153), while the pollen grains of 9311 plants were normal (Figure 3b,c). This result was consistent with gametophytic male sterility (Hu *et al*., 2014). Taken together, these observations indicated that the expression of the mitochondrial gene *orfH79* driven by pollen‐specific promoter *PG47* could inactivate rice pollen, ensuring the creation of the subsequent maintainer line with three‐element linkage expression.

### Construction of a linkage expression vector and development of the maintainer line 9311‐3B

**Figure 4:**
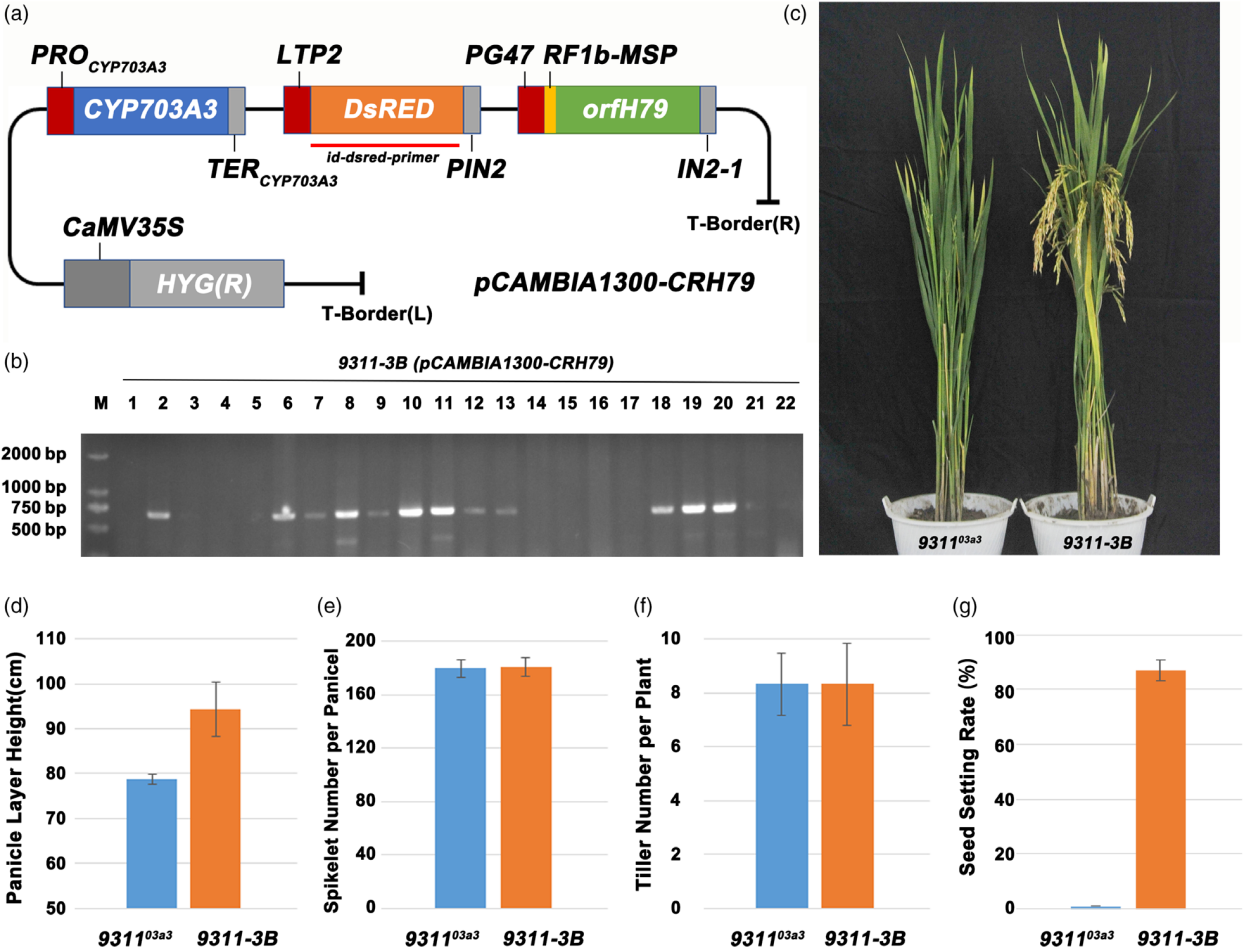
Construction and transformation of the three‐element linkage expression vector and phenotypic identification of the maintainer line. (a) Schematic diagram of the three‐element linkage expression vector *pCAMBIA1300‐CRH79*. (b) Identification of positive plants transformed by the three‐element linkage expression vector. (c) Observation of the plant architecture of the mutant *9311^03a3^* and the maintainer line 9311‐3B. (d–g) Statistics on panicle layer height, spikelet number per panicle, tiller number per plant and seed setting rate of the mutant *9311^03a3^* and the maintainer line 9311‐3B. [Colour figure can be viewed at wileyonlinelibrary.com]

**Figure 5:**
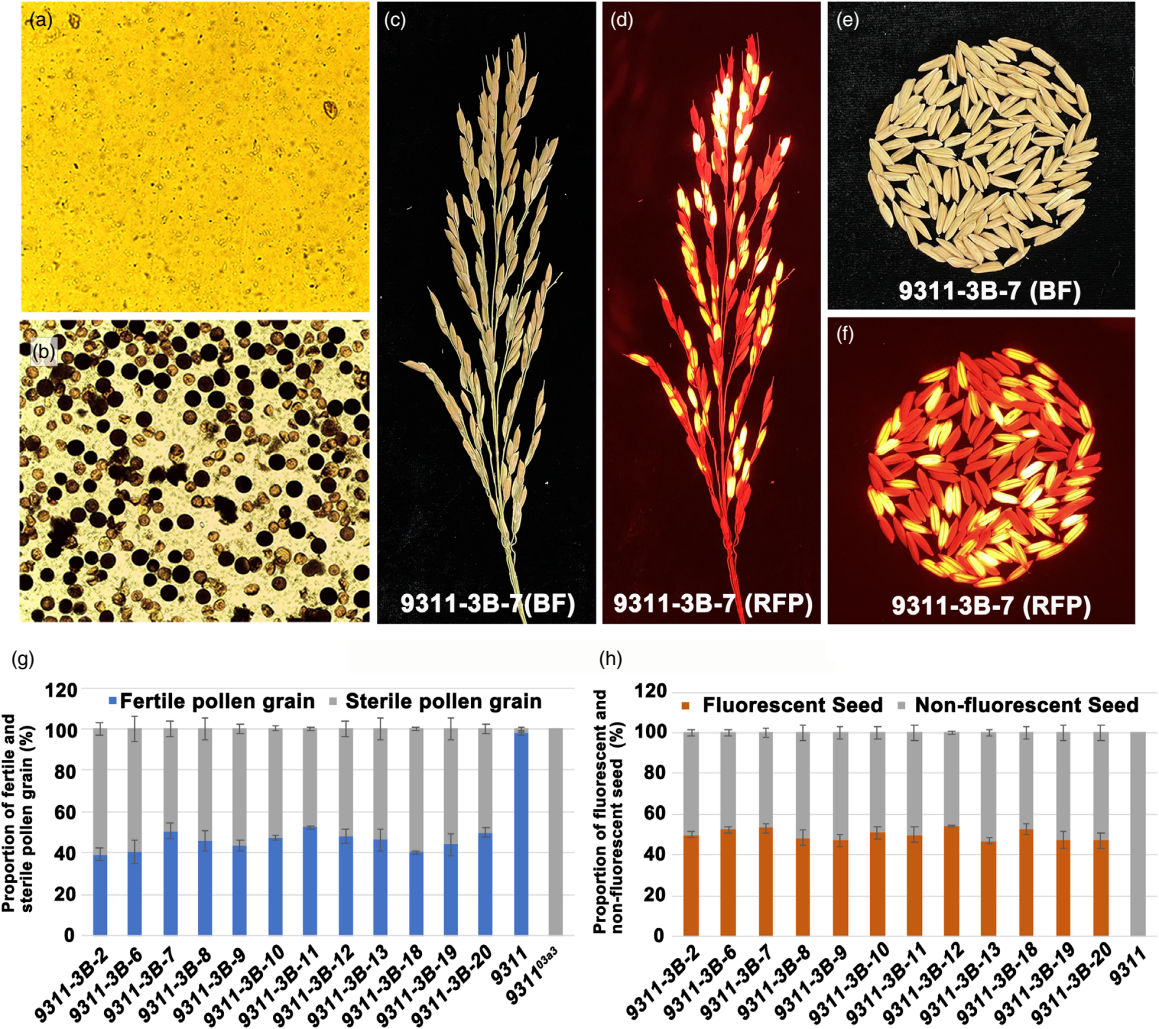
Pollen fertility and seed fluorescence of maintainer line 9311‐3B. (a, b) I_2_‐KI staining of pollen of the mutant *9311^03a3^* and the maintainer line 9311‐3B. (c, d) Panicle phenotype of the maintainer line 9311‐3B under bright field (BF) and red fluorescent field (RFP). (e, f) Grain phenotype of the maintainer line 9311‐3B under bright field (BF) and red fluorescent field (RFP). (g) The proportion of fertile pollen grain to sterile pollen grain in 12 independent 9311‐3B lines. (h) The proportion of fluorescent to nonfluorescent seeds in 12 independent 9311‐3B lines. [Colour figure can be viewed at wileyonlinelibrary.com]

Based on the strategy of the SPT system, the linkage expression vector *pCAMBIA1300‐CRH79* was constructed, containing the rice gene *CYP703A3*, selection marker gene *DsRed2* and pollen inactivation gene *orfH79*. In *pCAMBIA1300‐CRH79*, *CYP703A3* was driven by its native promoter, *DsRed2* was driven by the aleurone‐specific promoter *LTP2*, and *orfH79* was driven by the pollen‐specific promoter *PG47* (Figure 4a). Subsequently, the male‐sterile mutant *9311^03a3^* without foreign transgenic vectors was transformed with the linkage expression vector *pCAMBIA1300‐CRH79*. Twelve polymerase chain reaction (PCR)‐positive transformed plants were obtained as confirmed using specific primers (Figure 4B and Table S1). Moreover, the plant architecture, pollen fertility and other agricultural traits were checked. The panicle layer height was also rescued in 9311‐3B, suggesting that the restoration resulted from the loss of *CYP703A3* function (Figure 4c,d). Meanwhile, no significant change was found in the total spikelet number per plant and the tiller number per plant of 9311‐3B (Fig. 4e,f), while the seed setting rate of 9311‐3B was 87% (Figure 4g), and the results indicated that the linkage expression vector had no effects on these agricultural traits in rice. The pollen fertility I_2_‐KI staining of 12 positive independent lines showed that all lines exhibited 1:1 ratio of fertile and sterile pollen (Figure 5a,b and Figure 5g). In addition, the genetic segregation analysis of self‐pollination of 12 positive independent lines showed that all lines exhibited 1:1 ratio of fluorescent and nonfluorescent seeds (Figure 5c–f,h). These data indicated that by using the mitochondrial gene *orfH79,* the transgenic pollen grains were completely inactivated; moreover, a maintainer line of indica 9311 was successfully created using the linkage expression vector *pCAMBIA1300‐CRH79*. By self‐crossing the maintainer line, the sterile line 9311‐3A without the transgenic component and the maintainer line 9311‐3B with the transgenic component were produced simultaneously.

### Development of fluorescence sorting equipment for the third‐generation hybrid rice based on multispectral fluorescence imaging technology

One of the main advantages of the third‐generation hybrid system is enabling mechanized sorting of sterile and maintainer lines, dramatically reducing the cost of hybrid rice production. A fluorescence sorting equipment was created based on multispectral fluorescence imaging technology to establish a mechanized precision colour sorting platform for the third‐generation hybrid rice. The seeds were passed through the imaging system at a fixed interval under the combined actions of a vibrating plate and a conveyor belt. A laser with a central wavelength of 565 nm was used as the excitation light source to efficiently excite the weak fluorescent signal in the seeds after performing imaging experiments using multi‐excitation light and multispectral fluorescence with different types of seeds containing a fluorescent protein. A weak fluorescence image acquisition system was built with a high‐sensitivity fluorescence camera using a narrow‐band fluorescence filter with a central wavelength of 580 nm and the full width at half‐maximum of 14 nm, a lens of 8‐mm focal length and a 1‐inch complementary metal oxide semiconductor (CMOS) chip (Figure S3a). Three seeds were chosen from top to bottom, including those with strong fluorescence, weak fluorescence and none fluorescence, respectively, to check the efficiency (Figure S3b). The images were acquired using the fluorescence imaging system with an exposure time of 20 ms and 200 ms, respectively (Figure S3c,d). Observations showed that the system could highlight or only display the rice seeds containing fluorescence quickly. Subsequently, the acquired images were processed using the detection and classification system of rice seeds containing fluorescence based on a deep‐learning algorithm, and seeds containing fluorescence were detected and sorted. Then, sorting was conducted with 1000 seeds, resulting in the separation of 477 seeds with fluorescence and 523 seeds without fluorescence.

### Application of 9311‐3A for the third‐generation hybrid rice

**Figure 6:**
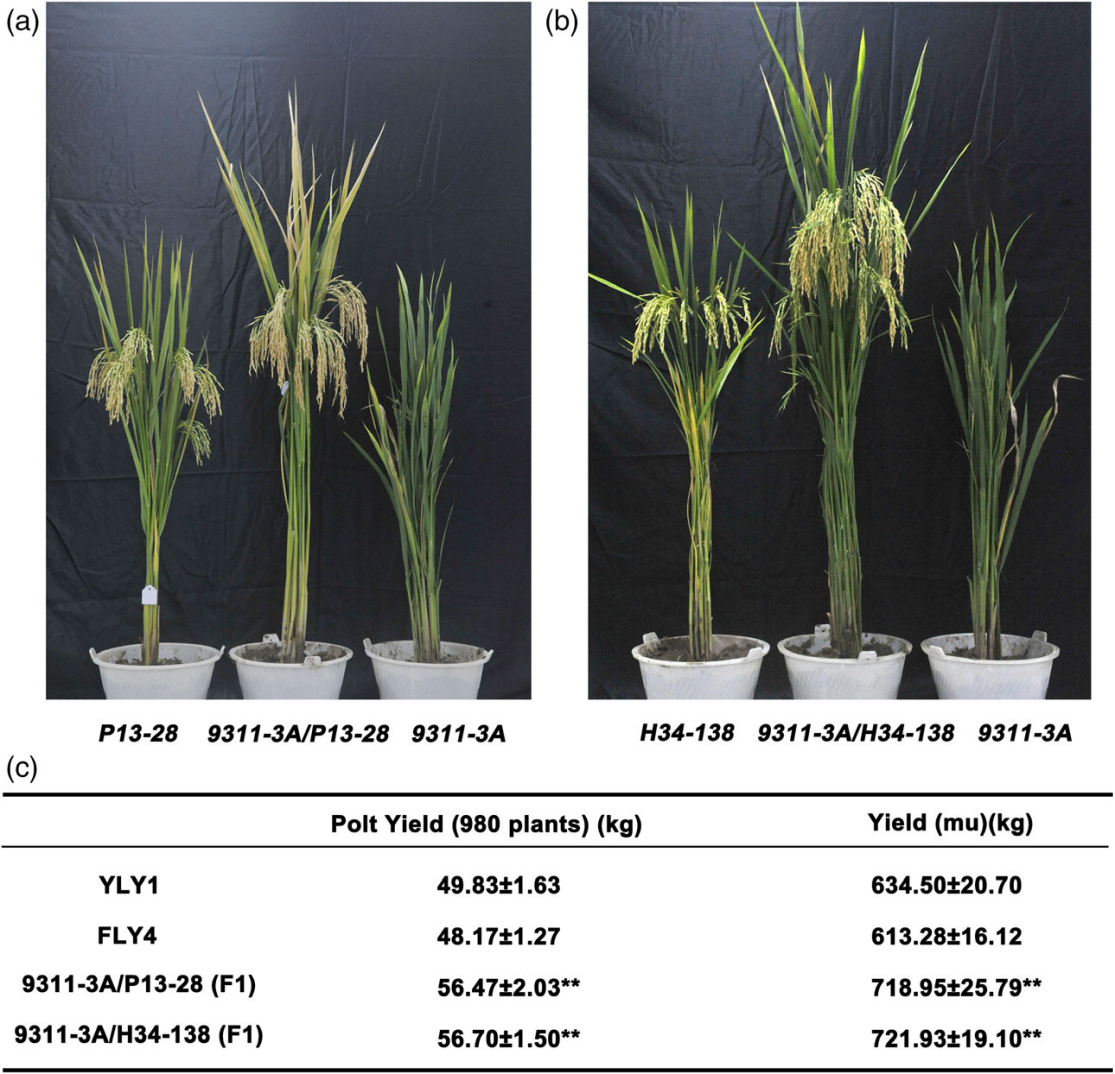
Observation on the plant‐type and agronomic traits of F1 generation by hybridization between the sterile line 9311‐3A and restorer lines P13‐28 and H34‐138. (a) Plant‐type observation of the hybrid F1 generation between the sterile line 9311‐3A and restorer lines P13‐28 and 9311‐3A/P13‐28. (b) Plant‐type observation of the hybrid F1 generation between the sterile line 9311‐3A and restorer lines H34‐138 and 9311‐3A/H34‐138. (c) Statistics on the plant plot yield and the yield (mu) of YLY1, FLY4, 9311‐3A/P13‐28 and 9311‐3A/H34‐138. [Colour figure can be viewed at wileyonlinelibrary.com]

Sterile line 9311‐3A was used as the female parent in the crosses to several elite inbreed lines to test its hybrid vigour potential and commercial prospects. The high‐quality varieties (restorer lines) P13‐28 and H34‐138 applied in production were used as the male parents for hybrid seed production. The hybridized combinations of 9311‐3A/P13‐28 and 9311‐3A/H34‐138 underwent field experiments with proper management. Furthermore, Y Liang You 1 (YLY1) and Feng Liang You 4 (FLY4) were planted and used as controls. Both these hybrids exhibited expected heterosis and ideal architecture with excellent performance (Figure 6a,b). The plot yield (980 plants) of 9311‐3A/P13‐28 and 9311‐3A/H34‐138 was approximately 56.47 kg and 56.70 kg, respectively, while that of YLY1 and FLY4 was approximately 49.83 kg and 48.17 kg, respectively (Figure 6c). The yield per mu of 9311‐3A/P13‐28 and 9311‐3A/H34‐138 was approximately 718.95 kg and 721.93 kg, respectively, increasing the production by more than 13% compared with the two control combinations (Figure 6c).

## Discussion

### Creation of novel indica GMS rice by knockout of *CYP703A3*

Since the utilization of CMS in rice 50 years ago, the three‐line hybrid rice has greatly contributed to food security in the world. The PTGMS system was developed 30 years ago with several pioneer varieties having been released that exhibited strong heterosis. CMS/Rf and PTGMS systems were named as the first‐ and second‐generation hybrid rice, respectively (Yuan, 2016). In line with this, the GMS and fluorescence marker selection systems have been termed as the third‐generation hybrid rice. The advantages of GMS rice, such as stable sterility, safe hybrid seed production and free for combination, could make up for the deficiencies of the CMS/Rf and PTGMS systems. However, the inability to propagate a pure male‐sterile line for commercial hybrid seed production severely blocked the application of the third‐generation hybrid rice. In this system, although the technology involved transgenics, only the maintainer line carried the transgenic elements; neither male‐sterile seeds nor hybrid seeds were transgenic for releasing.

In previous studies, researchers characterized *OsNP1* as a GMS gene, which encoded a putative glucose–methanol–choline oxidoreductase‐regulating tapetum degeneration and pollen exine formation (Chang *et al*., 2016). Besides *OsNP1*, several genic genes responsible for male fertility have been well characterized, including *Ms1*, *EAT1*, *TDR*, *Ms26, CYP704B2* and *CYP703A3* (Cigan *et al*., 2017; Fu *et al*., 2014; Li *et al*., 2010; Ono *et al*., 2018; Yang *et al*., 2019; Yang *et al*., 2014). The cytochrome P450 hydroxylase *CYP703A3* was highly conserved between japonica and indica rice which suggested that most common rice cultivars included functional *CYP703A3*. Moreover, the loss of function of *CYP703A3* led to complete pollen abortion in the variety 9311, in which complete restoration of fertility occurred by complementation. The *9311^03a3^* mutant exhibited complete pollen abortion, suitable flowering time and high stigma exsanguination. Because of incomplete heading, the mutant was lower than 9311 and the rate of florets that are wrapped inside the flag leaf is about 38%, but spraying gibberellins (GAs) can solve this problem. The findings showed that the GMS gene *CYP703A3* providing a guarantee for the creation of a breeding system of the recessive nuclear sterile line.

### CMS genes can be used as pollen killers

In the third‐generation hybrid rice system, the pollen‐lethality gene closely linked to the fertility restoration gene is another key element. The use of the pollen‐specific promoter to drive the expression of the pollen‐killer gene not only achieved the gametophytic segregation (1:1) of the progeny of the maintainer line but also prevented the escape of pollen containing the transgenic component, thus avoiding the transgenic safety event. Previously, DuPont–Pioneer devised SPT in a study using maize by the transformation of a male‐sterile mutant with a fertility restoration gene linked with the α‐amylase gene to disrupt the transgenic pollen and the *DsRed2* gene to mark the transgenic seeds. At present, *ZmAA1* could be used in this strategy (Wu *et al*., 2016). The α‐amylase protein encoded by this gene belongs to the family of glycosyl hydrolases, which catalyse and hydrolyse the polysaccharide molecule (1‐4)‐α‐D‐glycosidic bond. The expression of this gene was driven by the pollen‐specific promoter *PG47*.

This study tested the mitochondrial gene *orfH79*, which was responsible for HL‐CMS rice (Peng *et al*., 2010). Previous studies suggested that *orfH79* might be translated into a cytotoxic peptide that interacted with subunit P61 of the electron transport chain complex III, causing energy supply disorders and bursting of reactive oxygen species, eventually leading to pollen abortion (Wan *et al*., 2007; Wang *et al*., 2013; Yu *et al*., 2015). In this study, the cytotoxic peptide ORFH79 was fused with the MSP from *RF1b*. The resulting artificial ORFH79 was translocated into mitochondria, leading to mitochondrial dysfunction and, in turn, affecting the development of anther. The nuclear‐encoded ORFH79 is likely to function in mitochondria because the products of the *Rf5* and/or *Rf6* restorer genes only could cleave the *orfH79* transcripts at the post‐transcriptional level in the organelle (Hu *et al*., 2013; Hu *et al*., 2012; Huang *et al*., 2015). In this study, all the 12 positive independent maintainer lines achieved 1:1 ratio of fluorescent and nonfluorescent seeds. In addition, the maintainer line 9311‐3B was used to pollinate the sterile line 9311‐3A to determine the inactivation efficiency of pollen containing the linkage expression vector *pCAMBIA1300‐CRH79, and* the data indicated that the transgenic pollen grains were completely inactivated by using the mitochondrial gene *orfH79* (Table S2). Most characterized CMS genes have been shown to be mitochondrial chimeric orfs. Therefore, they may also be used as pollen killers for the third‐generation hybrid rice technology. The present study opened up another window for the future utilization of mitochondrial chimeric orfs such as WA352 in rice, urf13 in maize, orf138 in brassica, s‐pcf in petunia and orf552 in sunflower (Hu et al., 2014). It will greatly expand the repertoire of genes to be used as pollen killers for crop breeding.

### Development of the high‐efficient fluorescence seed‐sorting equipment in rice

Selection marker genes in the third‐generation hybrid rice system could accurately and efficiently sort the seeds of sterile and maintainer lines. At present, the most common selection marker was RFP, which was clearly visible to the naked eye under natural light and showed strong red fluorescence under the excitation light at 565 nm. In this study, the expression of the *DsRed2* gene was driven by the aleurone‐specific *LTP2* promoter, and the red seeds of the maintainer line containing the transgenic components and the colourless seeds of the sterile line containing no transgenic components were distinguished by colour sorting. Next, the efficiency and precision of the fluorescence sorting equipment were improved to guarantee no leakage of transgenic seeds so as to accelerate the industrialization of the third‐generation hybrid rice. In this study, the fluorescence sorting equipment was developed based on multispectral fusion, and a mechanized and precision sorting technology platform was established which can process 35 kg seeds per hour. Two modules of sorting were designed to achieve 100% accuracy. In the automatic‐sorting module, 99.99% accuracy was achieved in sorting seeds containing fluorescence, while in the reinspection module, 100% accuracy was achieved (Figure S3a). Consequently, the seeds of nontransgenic (9311‐3A) could be used for hybrid seed production to explore the heterosis, and the seeds of transgenic (9311‐3B) could be used for seed production of the maintainer and sterile lines simultaneously. The fluorescence sorting equipment is of great significance to the future commercialization of the third‐generation hybrid rice.

### High efficiency of seed production and potential for heterosis exploration

The high efficiency of seed production was quite important for hybrid crops. Nowadays, the male‐sterile and maintainer lines are sowed at different stages to produce seeds of the male‐sterile line, because of their different heading dates. Furthermore, the row ratio of the sterile and maintainer lines was 3:1 to improve seed production, and the maintainer lines should be cut away before harvesting the seeds of the sterile line. The seed production maximum was approximately 2.25–3.75 tons per hectare. In this study, the third‐generation hybrid rice system allowed for the maintainer and male‐sterile seeds to be segregated 1:1 at the same time. Therefore, crossing in the field for seed production of sterile lines was not needed. The lowest cost of seed production of sterile lines also could reach a yield of about 4.5–6 tons per hectare.

Ensuring global food security is an important task of agricultural science and technology. In most crops, hybrids exhibited strong heterosis, resulting in hybrid breeding technology being the most widely used and effective technology in crop breeding. In the last 30 years, two generations of hybrid rice, the CMS/Rf and PTGMS systems, have been released. The CMS sterile line only could be restored by restorer lines containing the restorer genes in nuclear genomes, limiting the exploitation of heterosis in rice. Also, the PTGMS sterile line was greatly affected by the external environment, making hybrid seed production quite vulnerable. In this study, the third‐generation hybrid rice breeding technology based on genetically engineered nuclear male‐sterile line was suggested to be one of the ideal ways to overcome the flaws of the CMS/Rf and PTGMS systems.

The potential for heterosis exploitation was tested by using 9311‐3A as the female parent and the common cultivar lines as the male parent, and the F1 hybrids were further tested. The results showed that the yield of these combinations for the third‐generation hybrid rice was significantly higher by more than 13% compared with the control varieties, showing strong heterosis. This suggested that the more free the common cultivars as male parents, the more the combination and the easier the exploration of strong heterosis. Consequently, the successful creation and application of this technology system could facilitate high quality, stable yield and efficient production, promoting green and sustainable development and ensuring world food security.

## Experimental procedures

### Plant materials and growth conditions

All the plants were grown in Hunan (summer) and Hainan (winter) provinces, China, under normal conditions. Agricultural traits, including panicle layer height, spikelet number, tiller number, heading date and stigma exsertion rate, were determined in both transgenic and control wild‐type plants. The panicle layer height was measured from the aboveground stem base to the tip of the panicle base in the mature stage. The tiller number and the grain number were determined in the mature stage and after harvesting. The pollen fertility was examined by I_2_‐KI staining as described in previous studies (Hu *et al*., 2012), and the flowering time of florets and the stigma exsertion rate of *9311^03a3^* and 9311 were investigated at the time of flowering. The daily flowering dynamic survey was carried out at the peak of flowering, the main panicle of 3 single plants were surveyed from 8:00 to 17:00, the number of opened florets during this period was recorded every 30 minutes, and this survey last for 3 consecutive days. The total number of flowering spikelets survey was carried out the main panicle of 3 single plants during flowering period.

### Creation of the male‐sterile mutant *9311^03a3^*

The CRISPR/Cas9 knockout vector pYLgRNA‐U3 and the plant expression vector pYLCRISPR/Cas9‐MH were gifted by Professor Liu Yaoguang from the South China Agricultural University. The gRNA of the target site of *CYP703A3* was inserted into pYLCRISPR/Cas9‐MH to obtain the CRISPR/Cas9 knockout construct of *CYP703A3* (Figure 1A). The construct was then introduced into 9311 calli by *Agrobacterium tumefaciens*‐mediated transformation. Specific primers were designed for PCR amplification, and the amplicon was sequenced for further analysis to check the mutation of *CYP703A3* (Table S1). Similarly, the presence of the hygromycin gene was also tested by PCR, which was absent in the mutant *9311^03a3^* (Table S1).

### Construction of the pollen‐killer system with HL‐CMS gene *orfH79*

Based on the studies on HL‐type hybrid rice, the *orfH79* gene expression element was synthesized by Qingke Biotechnology Co., Ltd., containing the promoter *PG47*, the *RF1b* MSP sequence and the terminator *IN2‐1*, and was introduced into *pCAMBIA1300*. Then, the construct *pCAMBIA1300‐orfH79* was transformed into 9311 calli mediated by *A. tumefaciens*. Transgenic calli and plantlets with hygromycin resistance were selected. The pollen fertility was examined by I_2_‐KI staining, as described in a previous study (Hu *et al*., 2012).

### Creation of the maintainer line 9311‐3B

The maintainer cassette vector *pCAMBIA1300‐CRH79* (*pCAMBIA1300‐CYP703A3‐DsRED‐orfH79*) was constructed in three steps. The *DsRED* expression element containing the promoter *LTP2*, *DsRed2* CDS and the terminator *PIN2* was first introduced into *pCAMBIA1300* to obtain *pCAMBIA1300‐DsRED*. Next, the *orfH79* expression element containing the promoter *PG47*, the gene *orfH79* and the terminator *IN2‐1* was introduced into *pCAMBIA1300‐DsRED* to obtain the recombinant vector *pCAMBIA1300‐DsRED‐orfH79*. Then, the *CYP703A3* expression element, including *CYP703A3*, with its native promoter and terminator, was cloned to be inserted into *pCAMBIA1300‐DsRED‐orfH79* and obtain a maintainer cassette vector, *pCAMBIA1300‐CRH79*. As no seeds were harvested from *9311^03a3^*, calli from *9311^03a3^* young panicles, which lacked the CRISPR/Cas9 vector, were induced. The mutant *9311^03a3^* was detected by sequencing, and the spikelets were taken in 3‐5 stages of development. The leaf sheaths were removed layer by layer and sterilized with 75% alcohol. Then, the exposed young spikes were placed on the Murashig and Skoog (MS) medium for induction. The vector *pCAMBIA1300‐CRH79* was transformed into the calli mediated by *A. tumefaciens*. Regenerated transgenic rice plants were grown and self‐crossed. Finally, 9311‐3B was obtained, and the agricultural traits were examined.

### Mechanical sorting of the transgenic and nontransgenic seeds

*DsRed2* derived from corals was used as the selection marker gene. The excitation centre wavelength of *DsRed2* was 558 nm, and the emission centre wavelength was 583 nm (Bevis and Glick, 2002). The seeds were irradiated with a continuous full‐band light source, and the wavelength of the excited fluorescence was measured with a fluorescence spectrometer. Then, the centre wavelengths of the excited and emitted lights of the seeds were determined. A high‐sensitivity fluorescence camera was used to capture the fluorescence image of rice seeds. The rice seed recognition method based on deep learning was used to detect the rice seeds in the captured image. If rice seeds with fluorescence were detected, an absorption mechanism was used to remove them.

### Creation of the combination of strong heterosis of the third‐generation hybrid rice

Two elite restorer cultivars (P13‐28 and H34‐138) were selected and crossed with the sterile line 9311‐3A to test the application potential of 9311‐3A. Two hybrid rice control varieties in regional yield trial YLY1 and FLY4 were used as controls. Then, the resulting four types of hybrid seeds were routinely planted in Hunan province, China, under proper management. The yield of the four hybrid populations was further analysed.

## Author contributions

L.L. and L.Y. designed the study and drafted the manuscript. S.S., T.W. and Y.L. performed the experiments, analysed the data and drafted the manuscript. J.H. and R.K. modified the draft. M.Q., Y.D., P.L., L.Z., H.D., C.L., D.Y., X.L. and D.Y. participated in performing the experiments. All authors reviewed and approved the manuscript for publication.

## Conflicts of interest

The authors declare no conflicts of interest.

## Supporting information


**Figure S1** Sequence list of CYP703A3 (DNA and protein) in 9311 and *9311^03a3^* background.

**Figure S2** Construction of complementary vector of male‐sterile mutant *9311^03a3^* and fertility testing.

**Figure S3** Model structure diagram of a colour sorter and detection of the intensity of fluorescent colour sorting.

**Table S1** Primers used in this study.

**Table S2** The percentage of red fluorescent seeds in the total hybrid seeds produced by the maintainer line 9311‐3B crossing with the sterile line 9311‐3A.
