# Supplementary material for: A novel strategy for creating a new system of third‐generation hybrid rice technology using a cytoplasmic sterility gene and a genic male‐sterile gene
Source: Plant Biotechnol J. 2020 Aug 27;19(2):251–60. doi: 10.1111/pbi.13457 (PMC7868973; doi:10.1111/pbi.13457)
Supplement: Supplementary file 1 — Figure S1 Sequence list of CYP703A3 (DNA and protein) in 9311 and 931103a3 background. [file PBI-19-251-s004.docx]

>CYP703A3 genomic sequence

TGACAGGAAAAGGCAGAGCACCAATCGTCAATAGTATAATCTTGCTCCTGAATGTCATCTATGCGTTACTTACAGAAATGTGCAAAAAATGAATTGCACTGAGCACATTGCTGAATAACAGTATGGAAGAGAACTTTTTTTTGGGTGAGCACATCTGAAGAGACTTTCTTTTATATTCATTCCCTCTTCTTTTTCCTTGGCATTTTCAGTGCTCTGCATCACACCATCTGTCATGTCAAGATCATGACGTTTATTATATGATGTTAGTTGAGATGTTGAACAATATAGATCAGCACAAGAATCATGACCAGTCTAATTCTGTAACCAATTCGCTTCAACTTTAGTCACTTGATTTGCTTTATCATAGAGCTTGATGAGATCCCATAAATAGGACTATAATTGCCATCTGTTTCTGTACCATGGATCCTTTTCTTCTTTCCATCATCTTATGCTCATGGATCTTTGTTGTAGTGTCCTGGAAAAAGCTCAATTGCATGAGGCGAAGGCTTCCACCTGGACCTCCAAGGTGGCCAATCTTTGGTAACCTTCTCCAGTTGAGCCCTCTTCCCCACAAAGACTTCGCTCGATTCTGCACCAAGTATGGCCCCCTGGTCTATCTTCGTCTTGGAACCATCGATGCCATCACCACTGATGACCCTGAAGTCATCCGTGAGATACTCATCCGGCAAGATGAGGTATTTGCTTCACGGCCTCGGACCTTGGCCGCTGTTCACCTTGCCTATGGGTGCGGTGATGTGGCCCTTGCTCCGCTGGGGCCGAACTGGAAGAGGATGAGGAGGGTTTGCATGGAGCACTTGCTGACAACCAAGCGGCTTGAATCTTTTGCTGCTCACCGAGCACTGGAGGCTGAGCACCTCTGTCAGTTTGTGTGGGCTAAAGCCCAGTCAGGGAAACCTGTGAATCTCAGAGAGGTTCTTGGGGCCTTCTCGATGAACAACGTGACAAGGATGTTGCTAGGGAAACAGTACTTTGGTCTGCAGTCAGCAGGCCCTGGTGAAGCAATGGAGTTCATGCACATCACCCATGAACTGTTCTGGCTGCTGGGCCTGATCTATCTTGGGGACTACTTGCCAGCCTGGAGGTGGCTTGATCCATATGGGTGTGAGAAGAAGATGAGGGAGGTTGAGAAGAAGGTGGACGACTTCCACCAGAAGATTATTGATGAGCACAGGAAGGCTAGGGAGGCCAAGAAGAGTGCATCCCTTGATGATGATAACAAAGAAGACATGGACTTTGTTGATGTACTGCTTTCTTTGCCCGGCGAGAACGGAAAAGAGCACATGGACGACGTCGAGATCAAAGCTTTGATGCAGGTATGTTTACTGTTTTTGTTATTCTAGTCTAGTTCTAAGTTCATTTGTTTTTTAACTAAGCCATCACTGTGTTTGTTGTTATAGTCTATTCTTGAAATTGAAATGATTTGCTAGCTGCCATTATGTTTGCAACAAATCTAGCCACGTTAATGTTATCTCCTTTGAGATTCAACCAATAGATGACTTGGTTCTTTTAAAGAAAATAATAAAAGGGAAGGTAATTAAAGATCTGTTTTCACAGCAGAGTTTTTGGTTCTTCTGTTGTTATGAGCTATGTTTGCTTTTCGGATTGAATTTATCTGTTACTTCTGGCAAACCATGTTATTTCATTTCTAGTGCTACATGTTGGAACCACAGTGAAGTGATGGACAGTACAAAGTACAAACTATCCTTTCTTGTCCTGGAGACTACAAGAAAATTAGAAGTAAAGAAATGATAGATTTTCTGAAAGTAAACTAGAAGAAAACATGTTGAACAACCTGTATTATTCTTCAACATTATGTGCATGCTAGCGCTACTAGTATACAACTGCATTTTTCAGTATCAGAAATCTTGCATAAGACATCAAATTAGTCATAAGCAATTCTGTTCTAAGTAGACAAAAACAAAACTAATCCATGTACACAACACAGGACATGATTGCTGCTGCCACCGACACATCATCGGTGACCAACGAGTGGGTGATGGCGGAGGTGATCAAGAACCCCCGGGTGCTCCGCAAGATCCAGGAGGAGCTCGACGGCGTCGTCGGCCGCGGCCGCATGGTGGCGGAGTCGGACCTCGGCCAGCTGACCTACCTCCGGTGCGTCGTCCGCGAGTCCTTCCGGATGCACCCGGCGGGGCCATTCCTGATCCCCCACGAGTCCCTGAAGCCGACGACGATCATGGGCTACGACATCCCGGCGCGGACGAGGATCTTCATCAACACCCACGCGCTCGGCCGGAACACCCGCATCTGGGACGACGTCGACGCGTTCCGGCCGGAGAGGCACCTGCCGGCATCGGCGGACGGCGGCCGGGTGGAGATCAGCCACCTGCCGGACTTCAAGATCCTCCCCTTCAGCGCCGGCAAGCGCAAGTGCCCCGGCGCGCCGCTCGGCGTCATCCTGGTGCTCATGGCGCTCGCCAGGCTCTTCCACTGCTTCGACTGGTCGCCGCCGGACGGCCTCCGCCCCGACGACATCGACACCCAGGAGGTGTACGGCATGACCATGCCCAAGGCCAAGCCGCTCGTCGCCGTCGCCACGCCGCGCCTGCCGCCGCAGATGTACGGTCGCCATGGCAAGCAAGTTTGAGCTAGGCGTCGTGTGGACAATAATGCAGACAGGAGAAGCCGATCGAGCTATGCAGCTTAACGTAATTAAGAGTGAATTAATTCTACATGTATCTATCAAATCAATCACACATGTAGACAGTGACGATGAATGCAAGTGCTGATGAATCAAAGAAATTAAGCATCAGCTGATGAATCTCACTTTCTTGCTGAATTCCATTCATTTCGTTCACACAGTAGGTTCTTTGAGACTAGATATCTTCTAGTACGTGCAAATAATACGCATCGTGTACTTCAAGGTTGTGATAAATATATTTCGTAGCAGAAGCTACTTAGCTCG

>CYP703A3 CDS sequence

ATGGATCCTTTTCTTCTTTCCATCATCTTATGCTCATGGATCTTTGTTGTAGTGTCCTGGAAAAAGCTCAATTGCATGAGGCGAAGGCTTCCACCTGGACCTCCAAGGTGGCCAATCTTTGGTAACCTTCTCCAGTTGAGCCCTCTTCCCCACAAAGACTTCGCTCGATTCTGCACCAAGTATGGCCCCCTGGTCTATCTTCGTCTTGGAACCATCGATGCCATCACCACTGATGACCCTGAAGTCATCCGTGAGATACTCATCCGGCAAGATGAGGTATTTGCTTCACGGCCTCGGACCTTGGCCGCTGTTCACCTTGCCTATGGGTGCGGTGATGTGGCCCTTGCTCCGCTGGGGCCGAACTGGAAGAGGATGAGGAGGGTTTGCATGGAGCACTTGCTGACAACCAAGCGGCTTGAATCTTTTGCTGCTCACCGAGCACTGGAGGCTGAGCACCTCTGTCAGTTTGTGTGGGCTAAAGCCCAGTCAGGGAAACCTGTGAATCTCAGAGAGGTTCTTGGGGCCTTCTCGATGAACAACGTGACAAGGATGTTGCTAGGGAAACAGTACTTTGGTCTGCAGTCAGCAGGCCCTGGTGAAGCAATGGAGTTCATGCACATCACCCATGAACTGTTCTGGCTGCTGGGCCTGATCTATCTTGGGGACTACTTGCCAGCCTGGAGGTGGCTTGATCCATATGGGTGTGAGAAGAAGATGAGGGAGGTTGAGAAGAAGGTGGACGACTTCCACCAGAAGATTATTGATGAGCACAGGAAGGCTAGGGAGGCCAAGAAGAGTGCATCCCTTGATGATGATAACAAAGAAGACATGGACTTTGTTGATGTACTGCTTTCTTTGCCCGGCGAGAACGGAAAAGAGCACATGGACGACGTCGAGATCAAAGCTTTGATGCAGGACATGATTGCTGCTGCCACCGACACATCATCGGTGACCAACGAGTGGGTGATGGCGGAGGTGATCAAGAACCCCCGGGTGCTCCGCAAGATCCAGGAGGAGCTCGACGGCGTCGTCGGCCGCGGCCGCATGGTGGCGGAGTCGGACCTCGGCCAGCTGACCTACCTCCGGTGCGTCGTCCGCGAGTCCTTCCGGATGCACCCGGCGGGGCCATTCCTGATCCCCCACGAGTCCCTGAAGCCGACGACGATCATGGGCTACGACATCCCGGCGCGGACGAGGATCTTCATCAACACCCACGCGCTCGGCCGGAACACCCGCATCTGGGACGACGTCGACGCGTTCCGGCCGGAGAGGCACCTGCCGGCATCGGCGGACGGCGGCCGGGTGGAGATCAGCCACCTGCCGGACTTCAAGATCCTCCCCTTCAGCGCCGGCAAGCGCAAGTGCCCCGGCGCGCCGCTCGGCGTCATCCTGGTGCTCATGGCGCTCGCCAGGCTCTTCCACTGCTTCGACTGGTCGCCGCCGGACGGCCTCCGCCCCGACGACATCGACACCCAGGAGGTGTACGGCATGACCATGCCCAAGGCCAAGCCGCTCGTCGCCGTCGCCACGCCGCGCCTGCCGCCGCAGATGTACGGTCGCCATGGCAAGCAAGTTTGA

>CYP703A3 Protein sequence

MDPFLLSIILCSWIFVVVSWKKLNCMRRRLPPGPPRWPIFGNLLQLSPLPHKDFARFCTKYGPLVYLRLGTIDAITTDDPEVIREILIRQDEVFASRPRTLAAVHLAYGCGDVALAPLGPNWKRMRRVCMEHLLTTKRLESFAAHRALEAEHLCQFVWAKAQSGKPVNLREVLGAFSMNNVTRMLLGKQYFGLQSAGPGEAMEFMHITHELFWLLGLIYLGDYLPAWRWLDPYGCEKKMREVEKKVDDFHQKIIDEHRKAREAKKSASLDDDNKEDMDFVDVLLSLPGENGKEHMDDVEIKALMQDMIAAATDTSSVTNEWVMAEVIKNPRVLRKIQEELDGVVGRGRMVAESDLGQLTYLRCVVRESFRMHPAGPFLIPHESLKPTTIMGYDIPARTRIFINTHALGRNTRIWDDVDAFRPERHLPASADGGRVEISHLPDFKILPFSAGKRKCPGAPLGVILVLMALARLFHCFDWSPPDGLRPDDIDTQEVYGMTMPKAKPLVAVATPRLPPQMYGRHGKQV*

>9311^03a3^-1 genomic sequence

TGACAGGAAAAGGCAGAGCACCAATCGTCAATAGTATAATCTTGCTCCTGAATGTCATCTATGCGTTACTTACAGAAATGTGCAAAAAATGAATTGCACTGAGCACATTGCTGAATAACAGTATGGAAGAGAACTTTTTTTTGGGTGAGCACATCTGAAGAGACTTTCTTTTATATTCATTCCCTCTTCTTTTTCCTTGGCATTTTCAGTGCTCTGCATCACACCATCTGTCATGTCAAGATCATGACGTTTATTATATGATGTTAGTTGAGATGTTGAACAATATAGATCAGCACAAGAATCATGACCAGTCTAATTCTGTAACCAATTCGCTTCAACTTTAGTCACTTGATTTGCTTTATCATAGAGCTTGATGAGATCCCATAAATAGGACTATAATTGCCATCTGTTTCTGTACCATGGATCCTTTTCTTCTTTCCATCATCTTATGCTCATGGATCTTTGTTGTAGTGTCCTGGAAAAAGCTCAATTGCATGAGGCGAAGGCTTCCACCTGGACCTCCAAGGTGGCCAATCTTTGGTAACCTTCTCCAGTTGAGCCCTCTTCCCCACAAAGACTTCGCTCGATTCTGCACCAAGTATGGCCCCCTGGTCTATCTTCGTCTTGGAACCATCGATGCCATCACCACTGATGACCCTGAAGTCATCCGTGAGATACTCATCCGGCAAGATGAGGTATTTGCTTCACGGCCTCGGACCTTGGCCGCTGTTCACCTTGCCTATGGGGTGCGGTGATGTGGCCCTTGCTCCGCTGGGGCCGAACTGGAAGAGGATGAGGAGGGTTTGCATGGAGCACTTGCTGACAACCAAGCGGCTTGAATCTTTTGCTGCTCACCGAGCACTGGAGGCTGAGCACCTCTGTCAGTTTGTGTGGGCTAAAGCCCAGTCAGGGAAACCTGTGAATCTCAGAGAGGTTCTTGGGGCCTTCTCGATGAACAACGTGACAAGGATGTTGCTAGGGAAACAGTACTTTGGTCTGCAGTCAGCAGGCCCTGGTGAAGCAATGGAGTTCATGCACATCACCCATGAACTGTTCTGGCTGCTGGGCCTGATCTATCTTGGGGACTACTTGCCAGCCTGGAGGTGGCTTGATCCATATGGGTGTGAGAAGAAGATGAGGGAGGTTGAGAAGAAGGTGGACGACTTCCACCAGAAGATTATTGATGAGCACAGGAAGGCTAGGGAGGCCAAGAAGAGTGCATCCCTTGATGATGATAACAAAGAAGACATGGACTTTGTTGATGTACTGCTTTCTTTGCCCGGCGAGAACGGAAAAGAGCACATGGACGACGTCGAGATCAAAGCTTTGATGCAGGTATGTTTACTGTTTTTGTTATTCTAGTCTAGTTCTAAGTTCATTTGTTTTTTAACTAAGCCATCACTGTGTTTGTTGTTATAGTCTATTCTTGAAATTGAAATGATTTGCTAGCTGCCATTATGTTTGCAACAAATCTAGCCACGTTAATGTTATCTCCTTTGAGATTCAACCAATAGATGACTTGGTTCTTTTAAAGAAAATAATAAAAGGGAAGGTAATTAAAGATCTGTTTTCACAGCAGAGTTTTTGGTTCTTCTGTTGTTATGAGCTATGTTTGCTTTTCGGATTGAATTTATCTGTTACTTCTGGCAAACCATGTTATTTCATTTCTAGTGCTACATGTTGGAACCACAGTGAAGTGATGGACAGTACAAAGTACAAACTATCCTTTCTTGTCCTGGAGACTACAAGAAAATTAGAAGTAAAGAAATGATAGATTTTCTGAAAGTAAACTAGAAGAAAACATGTTGAACAACCTGTATTATTCTTCAACATTATGTGCATGCTAGCGCTACTAGTATACAACTGCATTTTTCAGTATCAGAAATCTTGCATAAGACATCAAATTAGTCATAAGCAATTCTGTTCTAAGTAGACAAAAACAAAACTAATCCATGTACACAACACAGGACATGATTGCTGCTGCCACCGACACATCATCGGTGACCAACGAGTGGGTGATGGCGGAGGTGATCAAGAACCCCCGGGTGCTCCGCAAGATCCAGGAGGAGCTCGACGGCGTCGTCGGCCGCGGCCGCATGGTGGCGGAGTCGGACCTCGGCCAGCTGACCTACCTCCGGTGCGTCGTCCGCGAGTCCTTCCGGATGCACCCGGCGGGGCCATTCCTGATCCCCCACGAGTCCCTGAAGCCGACGACGATCATGGGCTACGACATCCCGGCGCGGACGAGGATCTTCATCAACACCCACGCGCTCGGCCGGAACACCCGCATCTGGGACGACGTCGACGCGTTCCGGCCGGAGAGGCACCTGCCGGCATCGGCGGACGGCGGCCGGGTGGAGATCAGCCACCTGCCGGACTTCAAGATCCTCCCCTTCAGCGCCGGCAAGCGCAAGTGCCCCGGCGCGCCGCTCGGCGTCATCCTGGTGCTCATGGCGCTCGCCAGGCTCTTCCACTGCTTCGACTGGTCGCCGCCGGACGGCCTCCGCCCCGACGACATCGACACCCAGGAGGTGTACGGCATGACCATGCCCAAGGCCAAGCCGCTCGTCGCCGTCGCCACGCCGCGCCTGCCGCCGCAGATGTACGGTCGCCATGGCAAGCAAGTTTGAGCTAGGCGTCGTGTGGACAATAATGCAGACAGGAGAAGCCGATCGAGCTATGCAGCTTAACGTAATTAAGAGTGAATTAATTCTACATGTATCTATCAAATCAATCACACATGTAGACAGTGACGATGAATGCAAGTGCTGATGAATCAAAGAAATTAAGCATCAGCTGATGAATCTCACTTTCTTGCTGAATTCCATTCATTTCGTTCACACAGTAGGTTCTTTGAGACTAGATATCTTCTAGTACGTGCAAATAATACGCATCGTGTACTTCAAGGTTGTGATAAATATATTTCGTAGCAGAAGCTACTTAGCTCG

>9311^03a3^-1 CDS sequence

ATGGATCCTTTTCTTCTTTCCATCATCTTATGCTCATGGATCTTTGTTGTAGTGTCCTGGAAAAAGCTCAATTGCATGAGGCGAAGGCTTCCACCTGGACCTCCAAGGTGGCCAATCTTTGGTAACCTTCTCCAGTTGAGCCCTCTTCCCCACAAAGACTTCGCTCGATTCTGCACCAAGTATGGCCCCCTGGTCTATCTTCGTCTTGGAACCATCGATGCCATCACCACTGATGACCCTGAAGTCATCCGTGAGATACTCATCCGGCAAGATGAGGTATTTGCTTCACGGCCTCGGACCTTGGCCGCTGTTCACCTTGCCTATGGGGTGCGGTGATGTGGCCCTTGCTCCGCTGGGGCCGAACTGGAAGAGGATGAGGAGGGTTTGCATGGAGCACTTGCTGACAACCAAGCGGCTTGAATCTTTTGCTGCTCACCGAGCACTGGAGGCTGAGCACCTCTGTCAGTTTGTGTGGGCTAAAGCCCAGTCAGGGAAACCTGTGAATCTCAGAGAGGTTCTTGGGGCCTTCTCGATGAACAACGTGACAAGGATGTTGCTAGGGAAACAGTACTTTGGTCTGCAGTCAGCAGGCCCTGGTGAAGCAATGGAGTTCATGCACATCACCCATGAACTGTTCTGGCTGCTGGGCCTGATCTATCTTGGGGACTACTTGCCAGCCTGGAGGTGGCTTGATCCATATGGGTGTGAGAAGAAGATGAGGGAGGTTGAGAAGAAGGTGGACGACTTCCACCAGAAGATTATTGATGAGCACAGGAAGGCTAGGGAGGCCAAGAAGAGTGCATCCCTTGATGATGATAACAAAGAAGACATGGACTTTGTTGATGTACTGCTTTCTTTGCCCGGCGAGAACGGAAAAGAGCACATGGACGACGTCGAGATCAAAGCTTTGATGCAGGACATGATTGCTGCTGCCACCGACACATCATCGGTGACCAACGAGTGGGTGATGGCGGAGGTGATCAAGAACCCCCGGGTGCTCCGCAAGATCCAGGAGGAGCTCGACGGCGTCGTCGGCCGCGGCCGCATGGTGGCGGAGTCGGACCTCGGCCAGCTGACCTACCTCCGGTGCGTCGTCCGCGAGTCCTTCCGGATGCACCCGGCGGGGCCATTCCTGATCCCCCACGAGTCCCTGAAGCCGACGACGATCATGGGCTACGACATCCCGGCGCGGACGAGGATCTTCATCAACACCCACGCGCTCGGCCGGAACACCCGCATCTGGGACGACGTCGACGCGTTCCGGCCGGAGAGGCACCTGCCGGCATCGGCGGACGGCGGCCGGGTGGAGATCAGCCACCTGCCGGACTTCAAGATCCTCCCCTTCAGCGCCGGCAAGCGCAAGTGCCCCGGCGCGCCGCTCGGCGTCATCCTGGTGCTCATGGCGCTCGCCAGGCTCTTCCACTGCTTCGACTGGTCGCCGCCGGACGGCCTCCGCCCCGACGACATCGACACCCAGGAGGTGTACGGCATGACCATGCCCAAGGCCAAGCCGCTCGTCGCCGTCGCCACGCCGCGCCTGCCGCCGCAGATGTACGGTCGCCATGGCAAGCAAGTTTGA

>9311^03a3^-1 Protein sequence

MDPFLLSIILCSWIFVVVSWKKLNCMRRRLPPGPPRWPIFGNLLQLSPLPHKDFARFCTKYGPLVYLRLGTIDAITTDDPEVIREILIRQDEVFASRPRTLAAVHLAYGVR*

>9311^03a3^-2 genomic sequence

TGACAGGAAAAGGCAGAGCACCAATCGTCAATAGTATAATCTTGCTCCTGAATGTCATCTATGCGTTACTTACAGAAATGTGCAAAAAATGAATTGCACTGAGCACATTGCTGAATAACAGTATGGAAGAGAACTTTTTTTTGGGTGAGCACATCTGAAGAGACTTTCTTTTATATTCATTCCCTCTTCTTTTTCCTTGGCATTTTCAGTGCTCTGCATCACACCATCTGTCATGTCAAGATCATGACGTTTATTATATGATGTTAGTTGAGATGTTGAACAATATAGATCAGCACAAGAATCATGACCAGTCTAATTCTGTAACCAATTCGCTTCAACTTTAGTCACTTGATTTGCTTTATCATAGAGCTTGATGAGATCCCATAAATAGGACTATAATTGCCATCTGTTTCTGTACCATGGATCCTTTTCTTCTTTCCATCATCTTATGCTCATGGATCTTTGTTGTAGTGTCCTGGAAAAAGCTCAATTGCATGAGGCGAAGGCTTCCACCTGGACCTCCAAGGTGGCCAATCTTTGGTAACCTTCTCCAGTTGAGCCCTCTTCCCCACAAAGACTTCGCTCGATTCTGCACCAAGTATGGCCCCCTGGTCTATCTTCGTCTTGGAACCATCGATGCCATCACCACTGATGACCCTGAAGTCATCCGTGAGATACTCATCCGGCAAGATGAGGTATTTGCTTCACGGCCTCGGACCTTGGCCGCTGTTCACCTTGCCT----GTGCGGTGATGTGGCCCTTGCTCCGCTGGGGCCGA

ACTGGAAGAGGATGAGGAGGGTTTGCATGGAGCACTTGCTGACAACCAAGCGGCTTGAATCTTTTGCTGCTCACCGAGCACTGGAGGCTGAGCACCTCTGTCAGTTTGTGTGGGCTAAAGCCCAGTCAGGGAAACCTGTGAATCTCAGAGAGGTTCTTGGGGCCTTCTCGATGAACAACGTGACAAGGATGTTGCTAGGGAAACAGTACTTTGGTCTGCAGTCAGCAGGCCCTGGTGAAGCAATGGAGTTCATGCACATCACCCATGAACTGTTCTGGCTGCTGGGCCTGATCTATCTTGGGGACTACTTGCCAGCCTGGAGGTGGCTTGATCCATATGGGTGTGAGAAGAAGATGAGGGAGGTTGAGAAGAAGGTGGACGACTTCCACCAGAAGATTATTGATGAGCACAGGAAGGCTAGGGAGGCCAAGAAGAGTGCATCCCTTGATGATGATAACAAAGAAGACATGGACTTTGTTGATGTACTGCTTTCTTTGCCCGGCGAGAACGGAAAAGAGCACATGGACGACGTCGAGATCAAAGCTTTGATGCAGGTATGTTTACTGTTTTTGTTATTCTAGTCTAGTTCTAAGTTCATTTGTTTTTTAACTAAGCCATCACTGTGTTTGTTGTTATAGTCTATTCTTGAAATTGAAATGATTTGCTAGCTGCCATTATGTTTGCAACAAATCTAGCCACGTTAATGTTATCTCCTTTGAGATTCAACCAATAGATGACTTGGTTCTTTTAAAGAAAATAATAAAAGGGAAGGTAATTAAAGATCTGTTTTCACAGCAGAGTTTTTGGTTCTTCTGTTGTTATGAGCTATGTTTGCTTTTCGGATTGAATTTATCTGTTACTTCTGGCAAACCATGTTATTTCATTTCTAGTGCTACATGTTGGAACCACAGTGAAGTGATGGACAGTACAAAGTACAAACTATCCTTTCTTGTCCTGGAGACTACAAGAAAATTAGAAGTAAAGAAATGATAGATTTTCTGAAAGTAAACTAGAAGAAAACATGTTGAACAACCTGTATTATTCTTCAACATTATGTGCATGCTAGCGCTACTAGTATACAACTGCATTTTTCAGTATCAGAAATCTTGCATAAGACATCAAATTAGTCATAAGCAATTCTGTTCTAAGTAGACAAAAACAAAACTAATCCATGTACACAACACAGGACATGATTGCTGCTGCCACCGACACATCATCGGTGACCAACGAGTGGGTGATGGCGGAGGTGATCAAGAACCCCCGGGTGCTCCGCAAGATCCAGGAGGAGCTCGACGGCGTCGTCGGCCGCGGCCGCATGGTGGCGGAGTCGGACCTCGGCCAGCTGACCTACCTCCGGTGCGTCGTCCGCGAGTCCTTCCGGATGCACCCGGCGGGGCCATTCCTGATCCCCCACGAGTCCCTGAAGCCGACGACGATCATGGGCTACGACATCCCGGCGCGGACGAGGATCTTCATCAACACCCACGCGCTCGGCCGGAACACCCGCATCTGGGACGACGTCGACGCGTTCCGGCCGGAGAGGCACCTGCCGGCATCGGCGGACGGCGGCCGGGTGGAGATCAGCCACCTGCCGGACTTCAAGATCCTCCCCTTCAGCGCCGGCAAGCGCAAGTGCCCCGGCGCGCCGCTCGGCGTCATCCTGGTGCTCATGGCGCTCGCCAGGCTCTTCCACTGCTTCGACTGGTCGCCGCCGGACGGCCTCCGCCCCGACGACATCGACACCCAGGAGGTGTACGGCATGACCATGCCCAAGGCCAAGCCGCTCGTCGCCGTCGCCACGCCGCGCCTGCCGCCGCAGATGTACGGTCGCCATGGCAAGCAAGTTTGAGCTAGGCGTCGTGTGGACAATAATGCAGACAGGAGAAGCCGATCGAGCTATGCAGCTTAACGTAATTAAGAGTGAATTAATTCTACATGTATCTATCAAATCAATCACACATGTAGACAGTGACGATGAATGCAAGTGCTGATGAATCAAAGAAATTAAGCATCAGCTGATGAATCTCACTTTCTTGCTGAATTCCATTCATTTCGTTCACACAGTAGGTTCTTTGAGACTAGATATCTTCTAGTACGTGCAAATAATACGCATCGTGTACTTCAAGGTTGTGATAAATATATTTCGTAGCAGAAGCTACTTAGCTCG

>9311^03a3^-2 CDS sequence

ATGGATCCTTTTCTTCTTTCCATCATCTTATGCTCATGGATCTTTGTTGTAGTGTCCTGGAAAAAGCTCAATTGCATGAGGCGAAGGCTTCCACCTGGACCTCCAAGGTGGCCAATCTTTGGTAACCTTCTCCAGTTGAGCCCTCTTCCCCACAAAGACTTCGCTCGATTCTGCACCAAGTATGGCCCCCTGGTCTATCTTCGTCTTGGAACCATCGATGCCATCACCACTGATGACCCTGAAGTCATCCGTGAGATACTCATCCGGCAAGATGAGGTATTTGCTTCACGGCCTCGGACCTTGGCCGCTGTTCACCTTGCCT----GTGCGGTGATGTGGCCCTTGCTCCGCTGGGGCCGAACTGGAAGAGGATGAGGAGGGTTTGCATG

GAGCACTTGCTGACAACCAAGCGGCTTGAATCTTTTGCTGCTCACCGAGCACTGGAGGCTGAGCACCTCTGTCAGTTTGTGTGGGCTAAAGCCCAGTCAGGGAAACCTGTGAATCTCAGAGAGGTTCTTGGGGCCTTCTCGATGAACAACGTGACAAGGATGTTGCTAGGGAAACAGTACTTTGGTCTGCAGTCAGCAGGCCCTGGTGAAGCAATGGAGTTCATGCACATCACCCATGAACTGTTCTGGCTGCTGGGCCTGATCTATCTTGGGGACTACTTGCCAGCCTGGAGGTGGCTTGATCCATATGGGTGTGAGAAGAAGATGAGGGAGGTTGAGAAGAAGGTGGACGACTTCCACCAGAAGATTATTGATGAGCACAGGAAGGCTAGGGAGGCCAAGAAGAGTGCATCCCTTGATGATGATAACAAAGAAGACATGGACTTTGTTGATGTACTGCTTTCTTTGCCCGGCGAGAACGGAAAAGAGCACATGGACGACGTCGAGATCAAAGCTTTGATGCAGGACATGATTGCTGCTGCCACCGACACATCATCGGTGACCAACGAGTGGGTGATGGCGGAGGTGATCAAGAACCCCCGGGTGCTCCGCAAGATCCAGGAGGAGCTCGACGGCGTCGTCGGCCGCGGCCGCATGGTGGCGGAGTCGGACCTCGGCCAGCTGACCTACCTCCGGTGCGTCGTCCGCGAGTCCTTCCGGATGCACCCGGCGGGGCCATTCCTGATCCCCCACGAGTCCCTGAAGCCGACGACGATCATGGGCTACGACATCCCGGCGCGGACGAGGATCTTCATCAACACCCACGCGCTCGGCCGGAACACCCGCATCTGGGACGACGTCGACGCGTTCCGGCCGGAGAGGCACCTGCCGGCATCGGCGGACGGCGGCCGGGTGGAGATCAGCCACCTGCCGGACTTCAAGATCCTCCCCTTCAGCGCCGGCAAGCGCAAGTGCCCCGGCGCGCCGCTCGGCGTCATCCTGGTGCTCATGGCGCTCGCCAGGCTCTTCCACTGCTTCGACTGGTCGCCGCCGGACGGCCTCCGCCCCGACGACATCGACACCCAGGAGGTGTACGGCATGACCATGCCCAAGGCCAAGCCGCTCGTCGCCGTCGCCACGCCGCGCCTGCCGCCGCAGATGTACGGTCGCCATGGCAAGCAAGTTTGA

>9311^03a3^-2 Protein sequence

MDPFLLSIILCSWIFVVVSWKKLNCMRRRLPPGPPRWPIFGNLLQLSPLPHKDFARFCTKYGPLVYLRLGTIDAITTDDPEVIREILIRQDEVFASRPRTLAAVHLACAVMWPLLRWGRTGRG*
