# Supplementary material for: A novel strategy for creating a new system of third‐generation hybrid rice technology using a cytoplasmic sterility gene and a genic male‐sterile gene
Source: Plant Biotechnol J. 2020 Aug 27;19(2):251–60. doi: 10.1111/pbi.13457 (PMC7868973; doi:10.1111/pbi.13457)
Supplement: Supplementary file 2 — Figure S2 Construction of complementary vector of male‐sterile mutant 931103a3 and fertility testing. [file PBI-19-251-s003.docx]

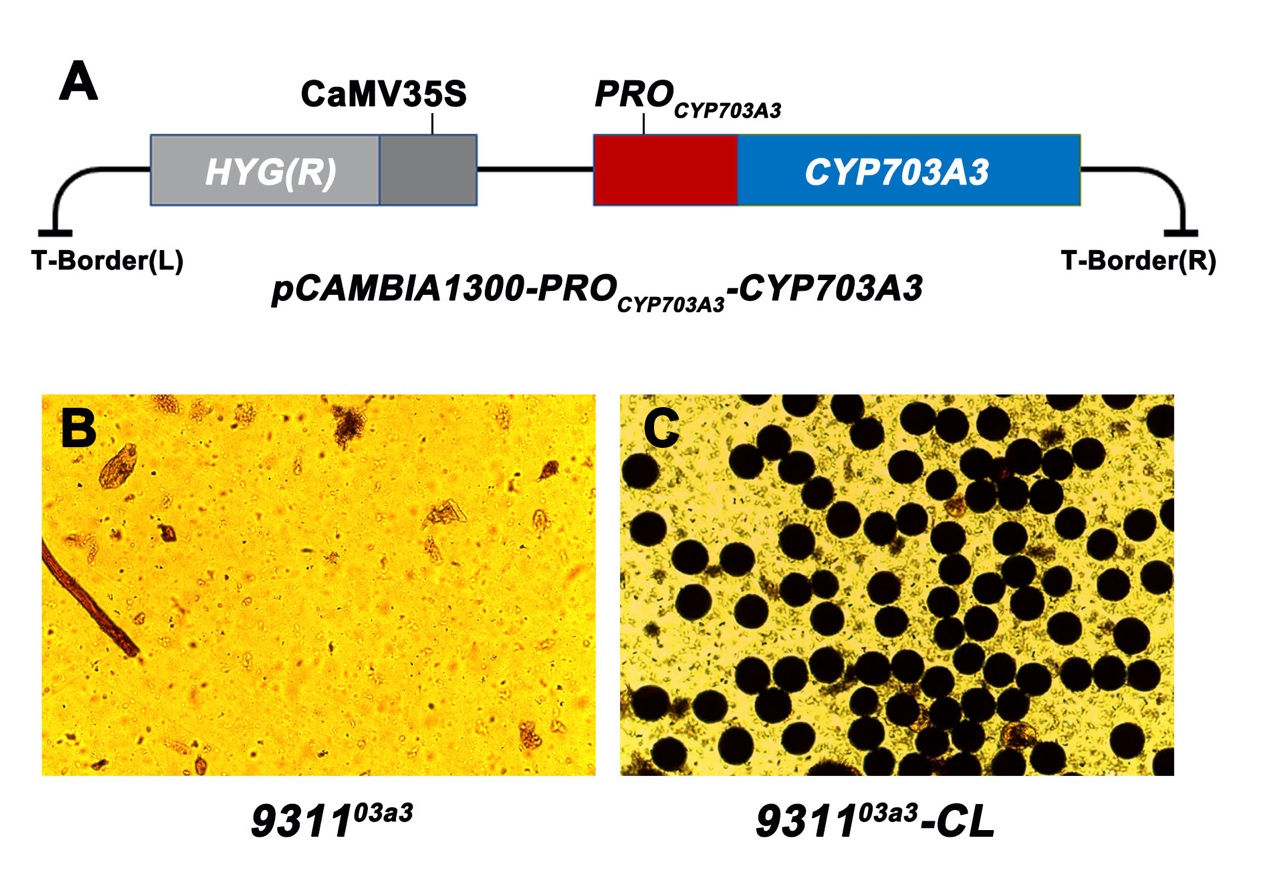


Figure S2 Construction of complementary vector of male sterile mutant *9311^03a3^* and its fertility testing

(A) Schematic diagram of *9311^03a3^* complementary vector;

(B) and (C) Anther fertility testing of *9311^03a3^* and complementary plant *9311^03a3^-CL*

To verify the fertility restorability and fertility restoration strength of *CYP703A3* in male sterile mutant *9311^03a3^*, a complementary vector of *CYP703A3* was constructed, and *9311^03a3^* was transformed, as shown in Figure S1A. The fertility testing of *9311^03a3^* and the complementary plant *9311^03a3^-CL* found that *9311^03a3^-CL* had a large number of pollens with normal fertility, , as shown in Figure S1B and S1C. It indicated that *CYP703A3* can restore the normal fertility of *9311^03a3^*, which ensured the creation of the subsequent breeding line with three-element linkage expression.
