# Supplementary material for: A novel strategy for creating a new system of third‐generation hybrid rice technology using a cytoplasmic sterility gene and a genic male‐sterile gene
Source: Plant Biotechnol J. 2020 Aug 27;19(2):251–60. doi: 10.1111/pbi.13457 (PMC7868973; doi:10.1111/pbi.13457)
Supplement: Supplementary file 3 — Figure S3 Model structure diagram of a colour sorter and detection of the intensity of fluorescent colour sorting. [file PBI-19-251-s002.docx]

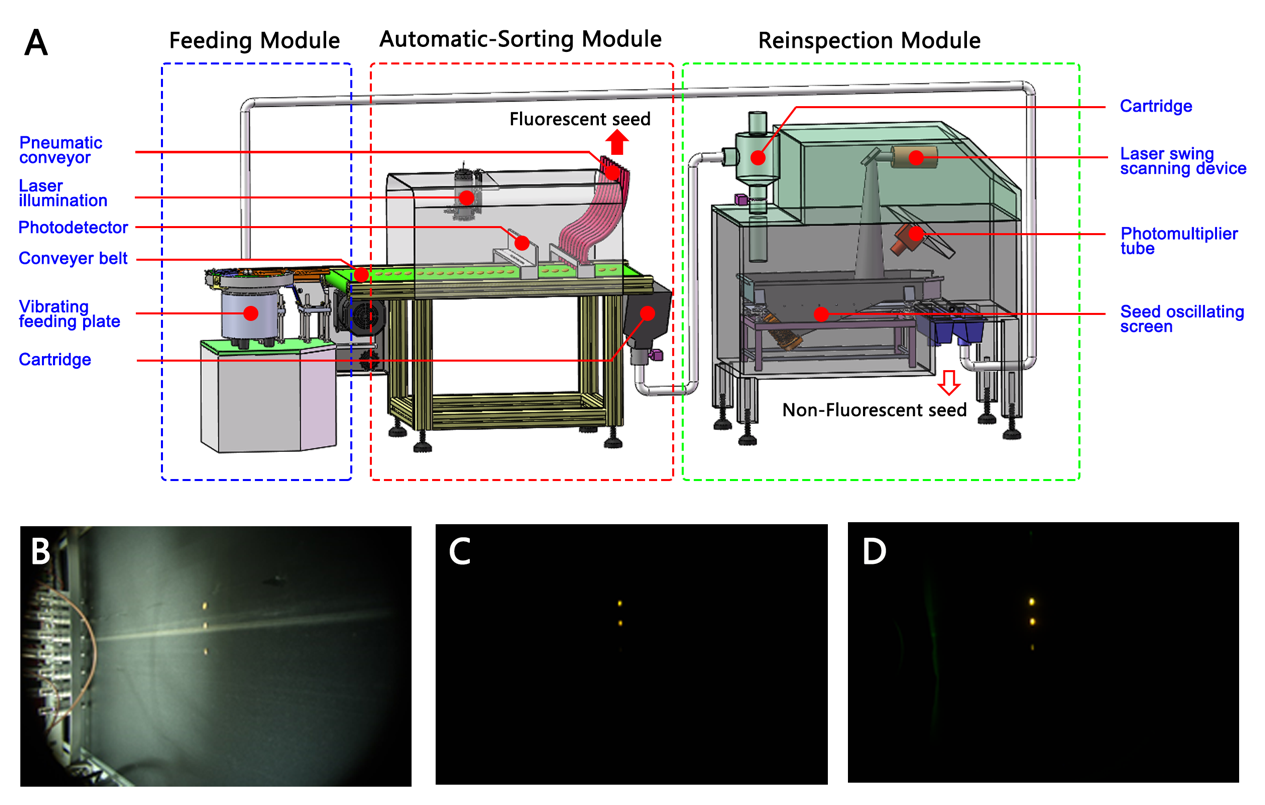


**Figure S3** Model structure diagram of a color sorter and detection of the intensity of fluorescent color sorting.

1. Model structure diagram of a color sorter.
2. Images of seeds under visible light.
3. Fluorescence image of seeds with 20-ms integration time.
4. Fluorescence image of seeds with 200-ms integration time.
