# Supplementary material for: A novel strategy for creating a new system of third‐generation hybrid rice technology using a cytoplasmic sterility gene and a genic male‐sterile gene
Source: Plant Biotechnol J. 2020 Aug 27;19(2):251–60. doi: 10.1111/pbi.13457 (PMC7868973; doi:10.1111/pbi.13457)
Supplement: Supplementary file 4 — Table S1 Primers used in this study. [file PBI-19-251-s005.docx]

Table S1 Primers used in this study.

F, forward primer; R, reverse primer

| Primer name Sequence 5’-3’ |
| --- |
| Primer for screening mutation of CYP703A3  GP3814-2757-F ATGGCCCCCTGGTCTATCTT  GP3814-2757-R GAAGGCCCCAAGAACCTCTC  Hyg-F ACCTGCCTGAAACCGAACTG  Hyg-R CTGCTCCATACAAGCCAACC  DsRed-F: ATGGCCTCCTCCGAGAACGT  DsRed-R: CTACAGGAACAGGTGGTGGC |
